# A Novel Formulation of Glucose-Sparing Peritoneal Dialysis Solutions with l-Carnitine Improves Biocompatibility on Human Mesothelial Cells

**DOI:** 10.3390/ijms22010123

**Published:** 2020-12-24

**Authors:** Francesca Piccapane, Mario Bonomini, Giuseppe Castellano, Andrea Gerbino, Monica Carmosino, Maria Svelto, Arduino Arduini, Giuseppe Procino

**Affiliations:** 1 Department of Biosciences, Biotechnologies and Biopharmaceutics, University of Bari, 70125 Bari, Italy; francesca.piccapane@uniba.it (F.P.); andrea.gerbino@uniba.it (A.G.); maria.svelto@uniba.it (M.S.); 2 Department of Medicine, G. d’Annunzio University of Chieti-Pescara, 66013 Chieti, Italy; mario.bonomini@unich.it; 3 Department of Emergency and Organ Transplantation, University of Bari, 70125 Bari, Italy; giuseppe.castellano@uniba.it; 4 Department of Sciences, University of Basilicata, 85100 Potenza, Italy; monica.carmosino@unibas.it; 5 Department of Research and Development, CoreQuest Sagl, Technopole, 6928 Manno, Switzerland; a.arduini@corequest.ch

**Keywords:** peritoneal dialysis, glucose-sparing, peritoneal dialysis solution, xylitol, mesothelium, l-carnitine

## Abstract

The main reason why peritoneal dialysis (PD) still has limited use in the management of patients with end-stage renal disease (ESRD) lies in the fact that the currently used glucose-based PD solutions are not completely biocompatible and determine, over time, the degeneration of the peritoneal membrane (PM) and consequent loss of ultrafiltration (UF). Here we evaluated the biocompatibility of a novel formulation of dialytic solutions, in which a substantial amount of glucose is replaced by two osmometabolic agents, xylitol and l-carnitine. The effect of this novel formulation on cell viability, the integrity of the mesothelial barrier and secretion of pro-inflammatory cytokines was evaluated on human mesothelial cells grown on cell culture inserts and exposed to the PD solution only at the apical side, mimicking the condition of a PD dwell. The results were compared to those obtained after exposure to a panel of dialytic solutions commonly used in clinical practice. We report here compelling evidence that this novel formulation shows better performance in terms of higher cell viability, better preservation of the integrity of the mesothelial layer and reduced release of pro-inflammatory cytokines. This new formulation could represent a step forward towards obtaining PD solutions with high biocompatibility.

## 1. Introduction

PD is an established home care, cost-effective kidney replacement therapy, for patients suffering from end-stage renal disease. PD uses the peritoneum as a biological dialysis membrane [1] and its blood depuration mode (dialytic exchange) is based on the exchange of solutes and removal of fluid from the blood in the peritoneal capillaries, through the infusion of 2 L of a PD solution into the peritoneal cavity via an implanted intra-abdominal catheter. Then, the effluent is drained after a dwell time (4 to 8 h) before fresh dialysate is re-infused either manually (Continuous Ambulatory PD; CAPD) with four exchanges in average per day or employing a cycler (Automated PD; APD) during the night (in average 8 h). The composition of the PD solution includes physiological concentrations of chloride, calcium, sodium, magnesium, a pH buffer (lactate and/or bicarbonate) and an osmotic agent to remove excess fluid from the patient via both the water-exclusive aquaporin and the small-pore, solute-coupled fluid pathways [2]. The main osmotic agent used in standard PD solution is glucose, due to its efficiency, low cost and acceptable safety profile.

PD offers several advantages as compared to hemodialysis, including better preservation of residual renal function, a more gradual and continuous solute and fluid clearance from the blood, minimal cardiac stress and similar survival rate [3]. However, PD is prescribed only to a minority of ESRD patients [1,4] because of the limited biocompatibility of the PD solutions, resulting in functional and/or structural injuries of mesothelial cells and peritoneal leukocytes [5]. The progressive damage to the PM is characterized by inflammation, neoangiogenesis, and fibrosis [6,7]. It is evidenced by fast peritoneal solute transport rate, progressive decline of UF capacity, which is associated with poor patient survival [8].

Severe transformation of the peritoneum, including progressive loss of the mesothelial cell layer, a massive increase in submesothelial thickness, and rapidly-progressing, severe peritoneal vasculopathy, is observed in patients under chronic PD with standard PD solutions [7]. PD solutions toxicity causes early and pronounced peritoneal inflammation, involving the invasion of the PM with macrophages and leucocytes, being the release of inflammatory cytokines another major driver of structural and functional deterioration [9,10]. Another key element of PM transformation is epithelial (mesothelial) to mesenchymal transition (EMT), featured by a migration of mesothelial cells into the submesothelium and transition to a myofibroblast cell type, and triggered by profibrotic and inflammatory cytokines [11,12]. Myofibroblasts can secrete inflammatory, proangiogenic, and profibrotic cytokines and extracellular matrix components [12]. The number of EMT cells in the submesothelium rapidly increases with the duration of PD treatment [9].

Though several potential factors have been claimed to be responsible for the poor biocompatibility of PD solutions, glucose is thought as the main culprit behind adverse events occurring locally and systemically during the PD lifetime of patients. Thus, several alternatives were examined over the years, but only two agents are currently used in glucose-free dialysates for clinical practice: icodextrin and amino acids (AA). It should be noted, however, that both solutions only replace up to 50% of daily glucose absorption [13]. Icodextrin is a glucose polymer derived from starch which allows a slow but sustained peritoneal UF and is indicated for use during a single long dwell per day [14]. A recent systematic review and meta-analysis showed that the use of icodextrin-containing PD solution, compared to a glucose-only PD regimen, improves peritoneal UF and reduces episodes of fluid overload [15]. However, this acidic PD solution has been associated with increased local and systemic inflammation [16,17]. AA-based solutions, with a pH of 6.6, have been developed in order to improve the dietary protein intake as well as the metabolic status of PD patients, though their use is limited to a single daily exchange due to the risk of acidosis and azotemia-related side effects [18]. The biocompatibility of AA-based solution remains uncertain, and experimental studies and findings in humans do not unanimously show improved peritoneal biocompatibility [19].

Reduced exposure of the PM to glucose is one of the key objectives of the current research in PD. A novel and tantalizing strategy is represented by the use of osmo-metabolic agents in the PD solutions [20,21]. This novel composition of PD solutions would ensure not only a reduction in the intraperitoneal glucose load without compromising UF, but also the independent mitigation of underlying metabolic disorders. l-carnitine has a molecular weight of 161.2 Da, is highly water-soluble, and is chemically stable in aqueous solutions, which suggest its suitability in PD fluid as a prototypical osmo-metabolic agent [22]. Several in vitro and in vivo investigations have indicated PD solution containing l-carnitine to be more biocompatible than standard glucose-based solutions [21,22]. In addition, our results in CAPD patients indicate that l-carnitine has potential use as a new osmotic agent in PD solution [23], and that l-carnitine-containing solution significantly improves insulin sensitivity and better maintains diuresis when compared to glucose-based solutions [24]. Of note, in all studies performed so far, the addition of l-carnitine to the PD solution has proven to be safe and well-tolerated by patients, without adverse events attributable to the treatment [22]. Osmo-metabolic agents may be used alone, or in combination, in order to maximize their therapeutic effects. l-carnitine and D-xylitol are a concrete example of such a combination. Xylitol (molecular weight 151.2 Da) is a five-carbon sugar alcohol involved in the pentose phosphate shunt, and has low glycemic properties [25]. In clinical trials [26], the use of xylitol-containing PD fluid proved to be safe, maintained peritoneal UF, and significantly improved glycemic control. Recently, we developed new PD solutions containing l-carnitine (1.24 mmol/L), xylitol (46 or 98.6 mmol/L), and a low amount of glucose (27.7 mmol/L) [21].

We previously reported that PD solutions containing xylitol and l-carnitine significantly improved endothelial cells viability in vitro, compared with conventional PD solutions [21].

In the present study, we compared the effects of this novel formulation of PD solutions to a wide number of commercial PD solutions, on human mesothelial cells. We demonstrated that this formulation improves cell viability, the integrity of mesothelial layer, and reduces the release of pro-inflammatory cytokines.

## 2. Results

### 2.1. Effect of Exposure to Different PD Solutions on Cell Viability and Transepithelial Electrical Resistance (TEER)

Human Mesothelial Cells (HMC) were cultured to confluence on extracellular matrix-coated inserts and exposed to the PD solutions only at the apical side, thus mimicking the condition of an 8 h PD dwell. Cell viability was evaluated after 8 h of exposure to different commercial PD solutions and compared to that of novel experimental solutions in which part of the glucose was substituted by xylitol and l-carnitine (glucose-sparing + l-carnitine). The same formulation without l-carnitine was also tested (glucose-sparing – l-carnitine). A positive control of cell viability was represented by cell monolayers exposed to culture medium at the apical side and a negative control was represented by cells exposed to 5 µM staurosporine to induce apoptosis.

**Figure 1:**
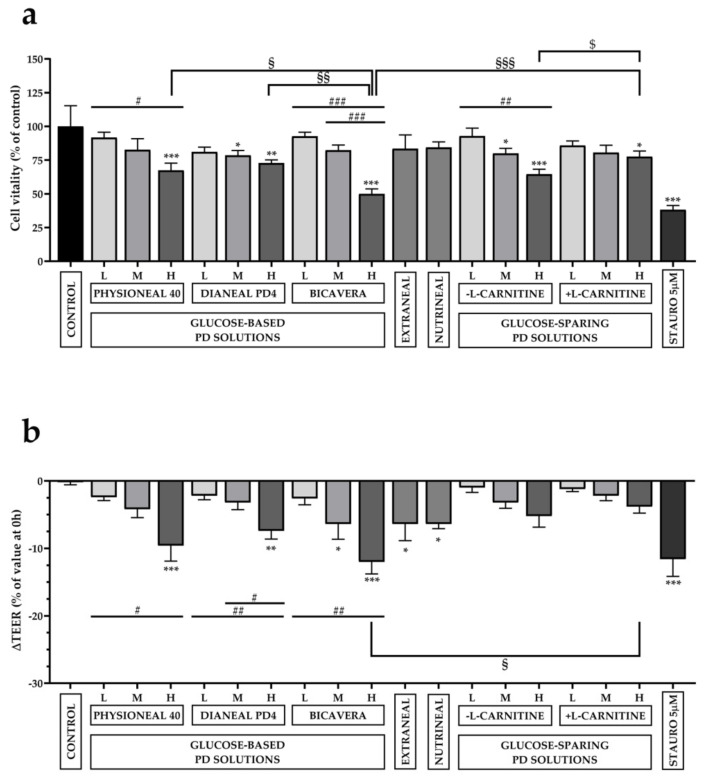
Effects of PD solutions on cell viability and TEER in HMC. Cells were exposed to low, medium and high (L, M, H) osmolarity glucose-based solutions (Physioneal 40^®^, Dianeal PD4^®^, BicaVera^®^), to glucose-free solutions (Extraneal^®^, Nutrineal^®^), and to low, medium and high (L, M, H) osmolarity glucose-sparing XyloCore solutions (± l-carnitine) for 8 h. (**a**) MTT assay was performed to evaluate cell viability. Cells incubated with Prigrow I medium were used as a control, and their cell viability set to 100%. Staurosporine (5 µM) served as a negative control of cell viability. (**b**) TEER was measured on each monolayer before and after incubation with PD solutions. Reductions of TEER (ΔTEER) were expressed as % of the value measured before exposure to the PD solutions. Significance was analyzed using a one-way ANOVA. Values are expressed as mean ± SEM. (*n* = 5). * *p* < 0.05; ** *p* < 0.01; *** *p* < 0.001 indicate statistical significance respect to control cells by one-way analysis of variance (ANOVA) with Dunnett’s multiple comparison test; # *p* < 0.05; ## *p* < 0.01; ### *p* < 0.001 values calculated by one-way analysis of variance (ANOVA) with Sidak’s multiple comparisons test, indicating how much the results differ within the same formulation as the glucose concentration varies. § *p* < 0.05; §§ *p* < 0.01; §§§ *p* < 0.001; values calculated by one-way analysis of variance (ANOVA) with Sidak’s multiple comparisons test, indicating how much the results differ from other treatment with the same concentration of glucose and same osmolarity. $ *p* < 0.05 values calculated in HMC incubated ± l-carnitine by *t*-test.

As reported in Figure 1a, cell viability decreased with increasing glucose concentrations (Low, Medium and High glucose concentration). As for Physioneal 40^®^, compared to control cells (set as 100% of cell viability), only the high-glucose formulation induced a strong, significant reduction of cell viability (67.38 ± 5.4%). As for Dianeal PD4^®^, both medium- and high-glucose solutions induced a significant reduction of cell viability (78.49 ± 3.62% and 72.89 ± 2.3%, respectively) compared to control. A dramatic reduction of cell viability was observed in cells exposed to high-osmolarity BicaVera^®^ PD solution (49.95 ± 3.72%). No statistically significant reduction of cell viability was observed in cells exposed to icodextrin-based (Extraneal^®^) and AA-based (Nutrineal^®^) solutions (83.46 ± 10.21% and 84.37 ± 4.12%, respectively). Strikingly, although in glucose-sparing solutions (- l-carnitine), a significant reduction of cell viability was observed in medium- and high-osmolarity solutions (79.97 ± 3.75% and 64.52 ± 3.71%, respectively), the presence of l-carnitine seemed to increase the biocompatibility of the solution. In fact, cell viability after exposure to high osmolarity solution (+ l-carnitine) was the highest (77.54 ± 4.21%), compared to all high osmolarity solutions and significantly higher compared to the same formulation without l-carnitine (64.52 ± 3.71%). This result might indicate an additional protective effect of l-carnitine on cell viability, at least after short-term exposure (8 h) to high osmolarity PD solutions.

Another interesting observation arising from these experiments is that high osmolarity BicaVera^®^, buffered at pH 7.4 by bicarbonate, is the PD solution with the lowest biocompatibility in this in vitro test.

As a direct index of mesothelial integrity, we measured the transepithelial electrical resistance (TEER) upon HMC exposure to all PD solutions reported above. TEER of HMC monolayers was measured before and 4 h after exposure to PD solutions at the apical side. Variations in TEER were expressed as % of the value measured before exposure to PD solutions. As reported in Figure 1b, in control cells, in which culture medium was maintained in the apical side, TEER did not significantly change during the 4 h interval. Instead, compared to control cells, TEER was significantly decreased in cells treated with high osmolarity Physioneal 40^®^ (−9.6 ± 2.29%) and Dianeal PD4^®^ (−7.4 ± 1.21%), medium and high osmolarity BicaVera^®^ (−6.4 ± 2.25% and −12 ± 1.79%, respectively). Also, exposure to glucose-free PD solutions Extraneal^®^ and Nutrineal^®^ induced a statistically significant reduction of TEER (−6.4 ± 2.46% and −6.4 ± 0.68%, respectively) compared to the control group. Strikingly, the glucose-sparing solutions (± l-carnitine), even at high osmolarity, induced only a slight, non-significant reduction of TEER compared to control. The presence of l-carnitine might produce an additional protective effect, although not statistically significant.

### 2.2. Effect of Exposure to Different PD Solution on Tight Junctions’ Integrity

**Figure 2:**
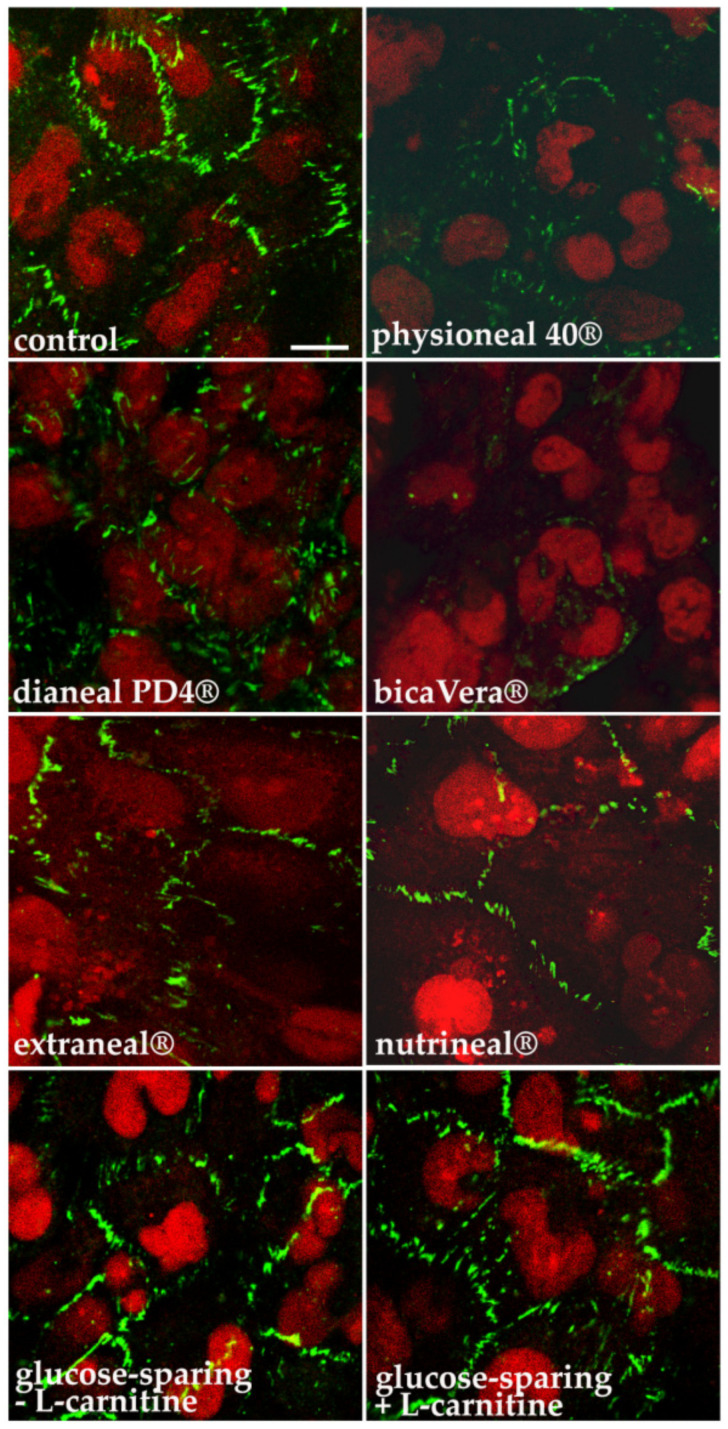
Immunofluorescence analysis of tight junction marker Zo-1 in HMC, grown on permeable supports and exposed for 8 h to different PD solutions at the apical side. HMC monolayers grown on permeable supports were incubated for 8 h at the apical side with high osmolarity glucose-based PD solutions (Physioneal 40^®^, Dianeal PD4^®^ and BicaVera^®^), glucose-free Extraneal^®^/Nutrineal^®^ and high osmolarity glucose-sparing PD solutions (± l-carnitine). Cells were immunostained with anti Zo-1 antibodies (green) and scanned by laser confocal microscopy. Nuclei were stained with propidium iodide (red). Scale bars = 10 μm.

Observation of cell monolayers under a bright-field microscope indicated that reduction of TEER, in monolayers exposed to high osmolarity PD solutions, was likely related to increased intercellular spaces without an evident detachment of cells from the culture inserts. We then measured the integrity of HMC epithelial barrier by evaluating the localization of the epithelial tight junction marker ZO-1 by immunofluorescence confocal analysis as reported in Figure 2.

HMC monolayers were either left in culture medium (control) or incubated for 8 h at the apical side with all the high osmolarity solutions used in the study plus Extraneal^®^ and Nutrineal^®^. Cells were then fixed and subjected to immunostaining with anti Zo-1 antibodies and nuclei were counterstained with propidium iodide. Confocal microscopy showed that ZO-1 was organized to form apical rings in control cells and became discontinuous after incubation with glucose-based solutions. In particular, the tight junction marker was strongly reduced after incubation with Physioneal 40^®^ and Dianeal PD4^®^ and almost disappeared after incubation with BicaVera^®^. A milder effect was induced by glucose-free solutions (Extraneal^®^ and Nutrineal^®^). Interestingly, treatment with glucose-sparing solutions did not alter ZO-1 expression that was apparently comparable to control cells.

### 2.3. Effect of Exposure to Different PD Solution on Pro-Inflammatory Cytokines

Next, we tested whether the exposure of HMC monolayers to different PD solutions could affect the release of different inflammatory cytokines. Considering the results obtained from the MTT test, TEER and immunofluorescence, we limited the analysis to high osmolarity PD solutions only.

**Figure 3:**
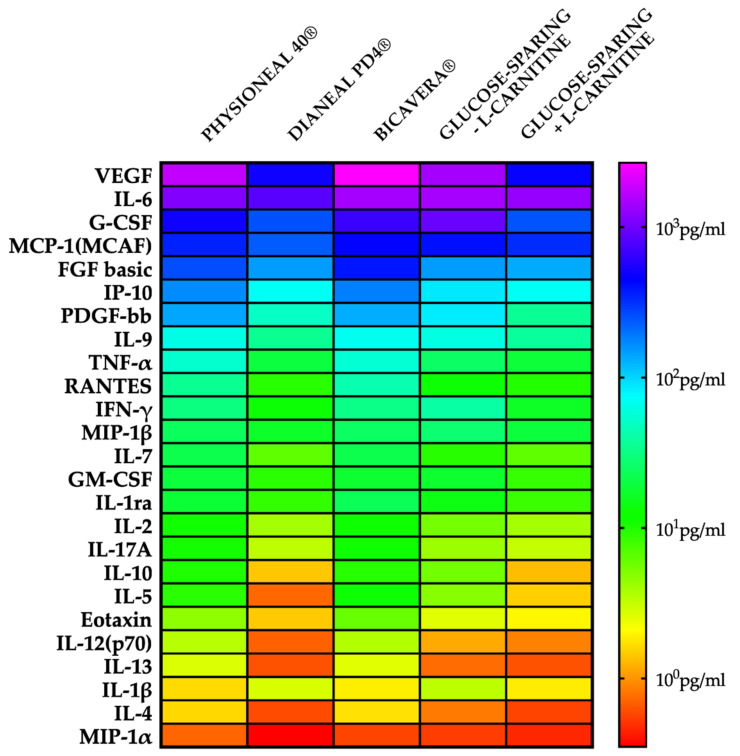
Cytokines, chemokines and growth factors released from the apical side by HMC incubated with high osmolarity PD solutions. A heatmap shows the result of a quantitative 27-plex Luminex assay. The concentration (pg/ml) increases as the color changes from red to violet.

As shown in the heatmap reported in Figure 3, we found that Physioneal 40^®^ and BicaVera^®^ induced a strong release of different pro-inflammatory cytokines compared to Dianeal PD4^®^ and glucose-sparing PD solutions. Interestingly, the apical release of IL-2, a pivotal growth factor regulating the proliferation of T lymphocytes [27], was very low in cells exposed to Dianeal PD4^®^ and glucose-sparing PD solutions. The release of IL-12p70, one of the key factors in the activation of dendritic cell response [28,29], was higher in cells exposed to Physioneal 40^®^/BicaVera^®^ than in cells exposed to glucose-sparing or Dianeal PD4^®^ solutions; the same effect was seen on the production of TNF-α, which induces maturation of dendritic cells. IL-17A was lower in HMC monolayers exposed to Dianeal PD4^®^ and glucose sparing PD solutions than in monolayers exposed to other PD solutions. Another important function of mesothelial cells is their ability to produce chemotactic factors for circulating leucocytes. As seen in Figure 3, we also found that HMC produced high levels of IP-10, a chemoattractant for monocyte/macrophages, T cells, NK cells and dendritic cells, upon exposure to Physioneal 40^®^/BicaVera^®^ [30]; on the contrary, glucose-sparing or Dianeal PD4^®^ solutions limited the release of IP-10 and RANTES being the latter another chemotactic factor to T cell, eosinophils and basophils [31].

Angiogenic growth factors such as FGF basic and VEGF [32] were released in relatively high levels in cells incubated with BicaVera^®^, at medium levels with Physioneal 40^®^, low levels with Dianeal PD4^®^ and even lower levels with glucose-sparing +l-carnitine solutions.

Finally, we also found that HMC could release high levels of growth factors in presence of Physioneal 40^®^/BicaVera^®^, such as GM-CSF (leucocytes), and PDGF-BB (fibroblast); interestingly, the glucose-sparing PD solutions or Dianeal PD4^®^ were capable to hamper the release of these growth factors that are involved in the inflammatory response during PD.

## 3. Discussion

Limited progress has been made in the last 50 years of peritoneal dialysis, regarding glucose replacement with a novel osmotic agent. Glucose-based solutions are still the most used and PD treatment still confers serious local and systemic toxicity.

In this work, we analyzed the response of human immortalized mesothelial cells grown as a monolayer to short-term exposure (8 h) to PD solutions with different composition. Specifically, we compared the effects induced by conventional glucose-based PD solutions (Physioneal 40^®^, Dianeal PD4^®^ and BicaVera^®^), glucose-free solutions (Extraneal^®^ and Nutrineal^®^) and a novel formulation of glucose-sparing solutions in which part of the glucose is substituted by xylitol with the addition of l-carnitine.

Here, for the first time, we aimed to recreate in vitro a mesothelium-like structure, using a cell line of human mesothelium, grown to confluence on porous cell culture inserts previously coated with a layer of an extracellular matrix. Cells were exposed to different PD solutions only at the apical side, as it happens during PD, and we evaluated cell viability, the integrity of the cell monolayer and the release of pro-inflammatory cytokines with a multidisciplinary approach. To the best of our knowledge, this is the first biocompatibility study in which mesothelial cells are exposed apically to PD solutions, thus recapitulating a condition more similar to PD in vivo and allowing transepithelial water and solutes fluxes.

A number of observations in both humans and animals indicated that hypertonic concentrations of glucose and glucose degradation products (GDP), along with acidic pH, are harmful to mesothelial cells function and viability [33].

Icodextrin-based PD solution may, in principle, protect PM from glucose and GDP, but there is some concern that icodextrin can induce a subclinical inflammatory response at both peritoneal and systemic level [33]. As for AA-based PD solutions, clinical observations and human studies have provided inconsistent results. Patients receiving AA-based solutions maintained a better nutritional state although the survival was comparable to controls receiving glucose-based solutions [34].

The fact that severe peritoneal damage is still observed in patients infused with low-GDP PD solutions, suggests that glucose per se has a deteriorating effect on the PM. A number of studies have shown direct adverse effects of high glucose concentrations on cellular function [35,36]. It has been demonstrated that high glucose in the conventional PD solutions induces cellular reactive oxygen species (ROS) in human peritoneal mesothelial cells and induces fibronectin expression, thus favoring the pathogenesis of peritoneal fibrosis [37]. Importantly, glucose may alter the expression of intercellular junctions within the mesothelium by decreasing the expression of junction-associated proteins such as *zonula occludens* protein 1 (ZO-1), E-cadherin, and β-catenin [38,39]. High osmolarity itself is also a key factor affecting the biocompatibility of PD solutions [40,41].

In this study, we used a new formulation that combines two osmotic agents, xylitol and l-carnitine, replacing a substantial part of glucose to improve the PD solution biocompatibility. We decided to leave some glucose in the PD solution as it is still a key nutrient for this category of patients often affected by malnutrition. The other reason was not to overexposed patients to xylitol as a very high concentration, over 150 g of intraperitoneal daily load [33], may have some adverse effects. However, it should be taken into account the current formulation of our glucose-sparing PD solution would get rid up to 80% of the glucose currently used in PD therapy as an osmotic agent.

By simulating an 8-h dwell, exposing the apical side of a HMC monolayer to PD solutions, we verified whether these solutions maintain a high percentage of cell viability. Interestingly, we observed that all the PD solutions currently used in clinical practice significantly reduce cell viability especially in the formulation with the highest osmolarity, with the worst performance shown by BicaVera which has the highest osmolarity (511 mOsm/l), compared to others (around 480 mOsm/l) and is buffered with bicarbonate. Among the glucose-sparing solutions, the addition of l-carnitine further increased cell viability of the highest osmolarity formulation. The beneficial effect provided by the addition of l-carnitine in PD solutions is known at the systemic level [22].

The new solutions were tested in cultured human umbilical vein endothelial cells (HUVECs) obtained from healthy and gestational diabetic mothers. In both cell types, the tested solutions did not induce cytotoxicity, nitro-oxidative stress, and inflammation caused by the neutral pH glucose-based PD solutions [21]. This suggests that a small amount of glucose may be maintained in the formulation of the PD solution, in order to take advantage of its UF ability and energy-providing potential.

However, the local effect on mesothelial cells has not been deeply investigated so far. An intriguing hypothesis that could explain the cytoprotective effect of l-carnitine in mesothelial cells exposed to high osmolar stress might involve the non-neuronal cholinergic system. We previously reported that human peritoneum mesothelial cells express the organic cation transporter OCTN1, implicated in the release of acetylcholine (Ach) outside the cell [42]. l-carnitine could be acetylated to acetylcarnitine in the mitochondria and provide acetyl groups for Ach biosynthesis which, in turn, would be transported outside the cell by OCTN1. Mesothelial cells expressα-7 nicotinic Ach receptors [43], and it has been demonstrated the existence of a cholinergic autocrine loop that can regulate cell growth [44,45]. Therefore, l-carnitine might boost Ach production and provide an anti-inflammatory effect [46], proliferation [44,45], and all the protective effects attributed to AChRs stimulation [47].

Another key parameter that we considered in our study is the effect of PD solutions on mesothelial integrity. Intact mesothelium provides in vivo resistance against solute permeation. Damage of intercellular junctions leads to an increase in the solute permeability. High glucose concentration, along with high osmolarity, damage intercellular junctions in human peritoneal mesothelial cells [38,48]. These changes are accompanied by a reduction in the TEER and increase in paracellular transport [49]. In this work, we provide compelling evidence that this novel formulation of PD solutions better preserves the integrity of mesothelial cells layer compared to conventional PD solutions. The drop in TEER was, in fact, particularly evident in HMC monolayers exposed to Physioneal 40^®^, Dianeal PD4^®^, and BicaVera^®^ and the effect seemed to be proportional to the glucose concentration. Interestingly, also Extraneal^®^ and Nutrineal^®^ induced a significant drop in TEER. Conversely, after exposure to glucose-sparing solutions, the drop in TEER was not statistically different from control monolayers, even when HMC were exposed to the high osmolarity solutions. Addition of l-carnitine showed a small additional ameliorative effect, although it was not statistically significant. Our glucose-sparing solutions were the ones with the best ability to preserve the integrity of mesothelial monolayer, as indicated by immunofluorescence analysis of Zo-1. Consistent with previous studies [38,50], the present research demonstrates that high concentrations of glucose in PD solutions induced mesothelial barrier disruption with a loss of epithelial tight junctions proteins such as ZO-1. Tight junctions’ integrity was preserved by exposure to our novel solutions and this might explain the effect on the TEER. Albeit obtained in an in vitro system, these results may have major implications in vivo. In fact, the high paracellular permeability is a feature of the so-called “high transporter” PD patients [51], in which the rapid dissipation of the osmotic gradient between the dialysis solution and the blood, leads to poor ultrafiltration. In this scenario, these new PD solutions, while preserving the integrity of the mesothelium, would guarantee better UF.

Moreover, the disruption of intercellular junctions and loss of apical–basolateral polarity in mesothelial cells trigger EMT, a process involving mesothelial cells transformation into fibroblast-like cells with increased migratory, invasive and fibrogenic features [52,53]. This novel formulation of PD solutions could prevent demesothelization, peritoneal hyperpermeability and a progressive reduction of UF. In addition, in this work, we evaluated the secretion of a panel of 27 cytokines, chemokines and growth factors released in response to exposure to all high osmolarity PD solutions. Interleukin-6 (IL-6) is a key player in modulating inflammation and can be secreted by mesothelial cells [54] in response to IL-1β [55]. In our assay, IL6 production seemed to be unaffected by the composition of the PD solutions. Mesothelial cells in vivo can produce TNFα in response to bacterial infection [56]. In our in vitro system, TNFα release is reduced by the novel PD solution formulation. Previous studies [57,58,59] demonstrated that exposure of the PM to PD solutions in mice increased the Th17 response and a consequent IL-17A production. Interestingly, IL-17A neutralization diminished peritoneal inflammation and fibrosis caused by chronic exposure to dialysis fluids in mice, thus suggesting that IL-17A could be a good therapeutic target to preserve the PM integrity in PD patients. In our hands, HMC released the lowest amount of IL-17A when exposed to the novel formulation of xylitol + l-carnitine solution. In response to pro-inflammatory mediators, mesothelial cells are able to secrete the chemokines RANTES and IP-10, recruiting leukocytes from blood vessels into the peritoneum, that is considered a diagnostic feature of peritonitis in PD patients [60,61]. We found decreased concentrations of these chemokines in response to exposure to the novel solutions. We also found that the release of VEGF is very low when the cells were exposed to novel PD solution formulations compared to conventional ones. This is very interesting since it has been demonstrated that mesothelial cells that underwent EMT are the main source of VEGF in PD patients [62]. Ogata and collaborators [63] demonstrated that exposure to high glucose levels caused a concentration-dependent increase of FGF basic mRNA expression and secretion by human peritoneal mesothelial cells, indicating that mesothelial cells are one of the peritoneal sources of this factor. Strikingly, in our study, the lowest level of FGF basic is secreted by HMC exposed to the novel PD solution. The role of PDGF-BB in PD is not fully understood. Patel and colleagues [64] demonstrated that overexpression of PDGF-BB in rat peritoneal tissue induces a “non-invasive” EMT process, characterized by angiogenesis without fibrosis. Anyhow, we found the lowest release of PDGF-BB by HMC cells exposed to our novel PD solution.

In conclusion, our data clearly demonstrate that mesothelial cells can release increased amounts of different pro-inflammatory and pro-fibrotic factors upon exposure to glucose-based PD solutions. We identified pro-inflammatory cytokines, leucocytes growth factors and chemokines activating several immune cells such as T lymphocytes and dendritic cells. In addition, the release of vascular and fibroblast growth factors could have a central role in the pathogenic alteration of peritoneum leading to irreversible changes in cellular responses to classical PD solutions. On the contrary, the use of our innovative PD solutions showed a limited capacity to induce the activation of the inflammasome in mesothelial cells, thereby maintaining proper cellular homeostasis.

Two clinical trials with the new osmo-metabolic formulation are under advanced development. FIRST (efficacy and safety assessments of a peritoneal dialysis solution containing glucose, xylitol and l-Carnitine compared to standard PD Solutions in CAPD) is an ongoing 1-month study (NCT04001036), whereas the ELIXIR trial (a study to Evaluate the efficacy and safety of Xylocore, a glucose sparing experimental solution for PD) is a planned international multicenter 6-month study, (NCT03994471). These studies will examine the safety, tolerability, and efficacy of the new PD solutions based on l-carnitine, xylitol and low-glucose not only on preservation of PM and residual kidney function, but also on underlying comorbidities able to increase cardiovascular risk.

The comparison of dialysis solutions currently used in clinical practice provides interesting points of discussion. In our experiments, BicaVera^®^ (neutral pH, bicarbonate buttered) appears to be less biocompatible than Physioneal 40^®^ (neutral pH and bicarbonate/lactate buffered) and Dianeal PD4^®^ (acidic pH and lactate buffered).

It should also be taken into account that the effect of the pH of peritoneal dialysates on peritoneal membrane morphology is still debated, with functional relevancy predominantly derived from experimental studies. Recent human studies by automated quantitative histomorphometry and molecular analyses on peritoneal tissue biopsies obtained from children with ESRD prior to and during maintenance PD with low GDP and neutral pH fluids have shown the same submesothelial fibrosis, loss of mesothelium, and advanced vasculopathy observed with conventional PD solutions (high GDP and acidic pH) [10]. Actually, a more evident increase in micro-vascular density correlating with an increase in small solute transport was seen with low GDP, neutral pH PD solutions when compared to conventional PD solutions. Though conclusions need to be drawn with caution, it has to be noted that clinical trials of low GDP, neutral pH PD solutions have failed to demonstrate a significant benefit with regards to peritoneal membrane function and UF capacity, suggesting that the presence of glucose as the main osmotic agent in PD therapy is the real culprit of the morphological changes observed in both low GDP, neutral pH and high GDP, acidic pH PD solutions [19,65,66].

## 4. Materials and Methods

### 4.1. Cell Culture

The Immortalized Human Mesothelial Cells-SV40 (HMC) were purchased from Applied Biological Materials (ABM, Richmond, BC, Canada). Cells were isolated from the human mesentery mesothelium and immortalized with recombinant lentiviruses carrying SV40 large T antigen. Cells were maintained in Prigrow I medium with 0.86 g/L glucose (cat. #TM001, Applied Biological Materials, Richmond, BC, Canada) supplemented with 10% fetal bovine serum, 100 i.u./mL penicillin, 100 μg/mL streptomycin, according to supplier indications. Cells were grown on 25 cm^2^ or 75 cm^2^ extracellular matrix-coated flasks (cat. #G299, Applied Biological Materials, Richmond, BC, Canada) at 37 °C in a humidified incubator and a 5% CO_2_ atmosphere. Culture medium was changed every 2–3 days and cells were subcultured when they became around 80% confluent. For all experiments, HMC were grown as monolayers on 24 wells permeable supports (cat. #353095 Transwell 0.4 μm pore size; Costar, Cambridge, MA, USA) previously coated with a thin layer of extracellular matrix (cat. #G422, Applied Biological Materials, Richmond, BC, Canada) at a density of 2.2 × 10^5^ cells/cm^2^. Experiments were generally performed five days after seeding when the cell monolayers reached high TEER.

### 4.2. Measurements of Transepithelial Electrical Resistance (TEER)

The transepithelial electrical resistance (TEER) was routinely measured with an Epithelial Voltohmmeter EVOM (World Precision Instruments, Sarasota, FL, USA) according to the manufacturer’s protocol. The EVOM system includes a pair of STX2 chopstick electrodes and allows non-destructive measurements of TEER. A TEER value used as blank was obtained measuring the electrical resistance across an insert without cells and then subtracted from the TEER values recorded across each monolayer. The final resistance value for unit area was obtained from each sample resistance multiplied for the surface area of the filter membrane (~0.3 cm^2^). The TEER across the HMC monolayers started to increase after 1 day of culture and the maximum value (~35 Ω∙cm^2^) was achieved between day 3 and 5, after which the TEER progressively decreased. The values of TEER obtained in our measurements are in agreement with previous reports on sheep and human mesothelium. TEER measured before exposure to different PD solutions was set as 100% for each monolayer. Variations of TEER were expressed as %.

### 4.3. PD Solutions Used in the Study

#### 4.3.1. Glucose-Based Solutions

- Physioneal 40^®^ (Baxter Healthcare Corp., Deerfield, IL, USA); pH = 7.4, bicarbonate and lactate-buffered; Low glucose (1.36%), Medium glucose (2.27%) and High glucose (3.86%);
- Dianeal PD4^®^ (Baxter Healthcare Corp., Deerfield, IL, USA); pH = 5–6.5, lactate-buffered; Low glucose (1.36%), Medium glucose (2.27%) and High glucose (3.86%);
- BicaVera^®^ (Fresenius Medical Care, Bad Homburg, Germany); pH = 7.4, bicarbonate-buffered; Low glucose (1.5%), Medium glucose (2.3%) and High glucose (4.25%).

#### 4.3.2. Glucose-Free Solutions

- Nutrineal^®^ (Baxter Healthcare Corp., Deerfield, IL, USA), pH = 5–6, lactate-buffered, 1.1% amino acids;
- Extraneal^®^ (Baxter Healthcare Corp., Deerfield, IL, USA), pH = 6.6, lactate-buffered, 7.5% icodextrin.

#### 4.3.3. Glucose-Sparing Solutions

- XyloCore, pH = 5.5, lactate-buffered; Low Strength: 0.7% Xylitol, 0.5% Glucose, and ± 0.02% l-carnitine, Medium Strength: 1.5% Xylitol, 0.5% Glucose, and ± 0.02% l-carnitine, High Strength: 2.0% Xylitol, 1.5% Glucose, and ± 0.02% l-carnitine

For detailed composition of each PD solution see Table 1.

### 4.4. Immunofluorescence and Confocal Microscopy

HMC were grown as monolayers on permeable filter supports for 5 days and then exposed to: high osmolarity Physioneal 40^®^, Dianeal PD4^®^, BicaVera^®^, Nutrineal^®^, Extraneal^®^, glucose-sparing solutions ± l-carnitine for 4 h at 37 °C in the incubator. After each treatment, monolayers were fixed in cold methanol (−20 °C) for 5 min, rinsed with phosphate-buffered saline (PBS) and blocked using 1% bovine serum albumin in PBS for 45 min at room temperature. After blocking, monolayers were incubated with the mouse antibody anti ZO1 (ZO1-1A12 cat. # 33-9100, dil. 1:1000; Thermo Fisher Scientific, Waltham, MA, USA), overnight at 4 °C in blocking buffer.

After three washes in PBS, cells were incubated with Alexafluor 488-conjugated secondary antibodies (Thermo Fisher Scientific, Waltham, MA, USA) for 1 h at room temperature, followed by washes with PBS and staining with propidium iodide (500 nM, Invitrogen, Thermo Fisher Scientific, Waltham, MA, USA) in SSC 2X (0.3 M NaCl, 0.03 M sodium citrate, pH 7.0) for 5 min. Monolayers were than washed several times with 2X SSC buffer and mounted in PBS/glycerol (1:1) containing 1% n-propylgallate, pH 8.0. Specimens were examined with a confocal laser-scanning fluorescence microscope (Leica SP5, Leica Microsystems, Milan, Italy).

### 4.5. MTT Assay

The effect of commercial PD solutions versus glucose-sparing solutions on HMC viability was assessed by the MTT assay. Cells were cultured as described above. On the day of the experiment, the culture medium in the apical side was replaced by 400 µl of different PD solutions, while the bottom of each well still contained 1ml of culture medium. Control cells maintained the culture medium in the apical compartment. As a negative control, we treated the cells with staurosporine, a protein kinase inhibitor commonly used to induce widespread apoptosis. Monolayers were exposed to PD solution for 4 h at 37 °C in the incubator. This allows us to mimic the in vivo condition during PD in which mesothelial cells are bathed by the PD solutions only at the apical side. TEER measurements were performed on each monolayer before and after incubation with each PD solution. Following incubation, 40 μL of tetrazolium MTT (5 mg/mL) (3-(4, 5-dimethylthiazolyl-2)-2, 5 diphenyltetrazolium bromide) were added in the apical compartment and cells were incubated at 37 °C for an additional 4 h. During the reaction, the yellow tetrazolium salt MTT is converted to purple formazan crystals by intracellular reducing equivalents produced by metabolically active cells. Subsequently, 440 μL of acidic isopropanol (0.04 N HCl in isopropanol) were added to each insert and mixed thoroughly to dissolve the generated formazan crystals. The spectrometric absorbance value of the wells was read at 595 nm and 620 nm using a microplate reader (Bio-Rad, Hercules, CA, USA). Cells viability upon different PD solutions was expressed as the percentage of control cells.

### 4.6. Cytokines Profiling Assay

A Luminex assay (#M500KCAF0Y, Bio-Plex Pro 27 Plex Human Cytokine kit, Bio-Rad Laboratories, Hercules, CA, USA) enabling the simultaneous measurement of 27 secreted cytokines in the conditioned media of HMC was performed. The panels included the following 27 biomarkers:

Cytokines: IL-1β, IL-1 receptor antagonist (IL-1Ra), IL-2, IL-4, IL-5, IL-6, IL-7, IL-8, IL-9, IL-10, IL-12 (p70), IL-13, IL-15, IL-17, IFN-γ, tumor necrosis factor alpha (TNF-α);

Chemokine: Eotaxin, interferon-induced protein 10 (IP-10), monocyte chemoattractant protein 1 (MCP-1), macrophage inflammatory protein 1 alpha and 1 beta (MIP-1α and MIP-1β), regulated on activation T cell expressed and secreted (RANTES);

Growth factors: fibroblast growth factor (FGF Basic), granulocyte colony-stimulating factor (G-CSF), granulocyte-macrophage CSF (GM-CSF), platelet-derived growth factor BB (PDGF-BB), vascular endothelial growth factor (VEGF).

Cells were cultured as described above. On the day of the experiment, the apical culture medium was replaced by different PD solutions as previously described and the cell monolayers incubated for 8 h. Apical media from each well were collected; FBS was added to apical samples at same concentrations of the culture medium; samples were centrifuged at 1000× *g* for 15 min at 4 °C to remove any precipitate and stored at −80 °C until use. Briefly, a mixture of recombinant analyte standards was diluted in culture medium and a standard curve composed of 8 points was prepared. Standards, blanks, and samples (50 μL) were added to a test 96 well plate containing antibodies that were chemically attached to fluorescent-labeled microbeads and were incubated in the dark under shaking (900 rpm for 30 min) at room temperature. After washing steps, the biotinylated detection antibodies were added to each well, and the plate was incubated in the dark for 30 min at RT with continuous shaking. The plate was washed again, and streptavidin-phycoerythrin (50 μL) was added to each well and incubation continued in the dark for 10 min at RT with shaking. Assay buffer (125 μL) was added to each well of the plate before being read on a BioPlex Magpix Multiplex Reader (Bio-Rad Laboratories, Hercules, CA, USA). Each sample was analyzed in duplicate and the data automatically analyzed and processed using Bio-Plex Manager 6.0 software (Bio-Rad Laboratories, Hercules, CA, USA).

### 4.7. Statistical Analysis

All MTT and TEER experiments were repeated at least five times, and the results are presented as mean ± standard error of the mean (SEM). Statistical analysis was performed by one-way analysis of variance (ANOVA) with Dunnett’s test (comparing all groups to control) or with Sidak’s test (for multiple comparisons) to compare the effects of the different PD solutions. A two-tailed unpaired t-test was used to compare data sets in glucose-sparing solutions with and without l-carnitine using GraphPad Prism version 8.0 for Windows (GraphPad Software, San Diego, CA, USA). Significance was defined as a *p*-value < 0.05.

## 5. Conclusions

Our results indicate that a new formulation of glucose-sparing PD solutions with xylitol and l-carnitine might represent a step forward in the search of a more biocompatible dialytic solution in PD.

Although obtained in an in vitro system, these results indicate that the main drawbacks associated with conventional glucose-based PD solutions, namely the loss of mesothelial cells, fibrosis, vasculopathy, and inflammation, might be prevented by those solutions. Clinical trials are under advanced development. Nonetheless, the mechanism through which l-carnitine plays a protective role of on the mesothelium deserves further investigation at subcellular level.

## Figures and Tables

**Table 1:** Composition of PD solutions.

	GLUCOSE- BASED PD SOLUTIONS								
	Physioneal 40^®^			Dianeal PD4^®^			BicaVera^®^		
mMol/L	LOW	MEDIUM	HIGH	LOW	MEDIUM	HIGH	LOW	MEDIUM	HIGH
**Sodium**	132	132	132	132	132	132	134	134	134
**Calcium**	1.25	1.25	1.25	1.25	1.25	1.25	1.75	1.75	1.75
**Magnesium**	0.25	0.25	0.25	0.25	0.25	0.25	0.5	0.5	0.5
**Chloride**	95	95	95	95	95	95	104.5	104.5	104.5
**Glucose**	75.5	126	214	75.5	126	214	83.25	126.1	235.9
**Xylitol**	-	-	-	-	-	-	-	-	-
**Lactate**	15	15	15	40	40	40	-	-	-
**Bicarbonate**	25	25	25	-	-	-	34	34	34
**Icodextrin**	-	-	-	-	-	-	-	-	-
**Aminoacids**	-	-	-	-	-	-	-	-	-
**l-carnitine**	-	-	-	-	-	-	-	-	-
**Osmolarity** ***(mOsmol/L)***	344	395	483	344	395	483	358	401	511
**pH**	7.4	7.4	7.4	5–6.5	5–6.5	5–6.5	7.4	7.4	7.4
	**GLUCOSE- FREE** **PD SOLUTIONS**			**GLUCOSE-SPARING PD SOLUTIONS**					
	**Extraneal^®^**		**Nutrineal^®^**	**− l-CARNITINE**			**+ l-CARNITINE**		
**mMol/L**	**Icodextrin 7.5%**		**Amino acids 1.1%**	**LOW**	**MEDIUM**	**HIGH**	**LOW**	**MEDIUM**	**HIGH**
**Sodium**	133		132	134	134	134	134	134	134
**Calcium**	1.75		1.25	1.75	1.75	1.75	1.75	1.75	1.75
**Magnesium**	0.25		0.25	0.5	0.5	0.5	0.5	0.5	0.5
**Chloride**	96		105	103.5	103.5	103.5	103.5	103.5	103.5
**Glucose**	-		-	27.7	27.7	83	27.7	27.7	83
**Xylitol**	-		-	46	98.6	125	46	98.6	125
**Lactate**	40		40	35	35	35	35	35	35
**Bicarbonate**	-		-	-	-	-	-	-	-
**Icodextrin**	7.5 (%)		-	-	-	-	-	-	-
**Aminoacids**	-		87.16	-	-	-	-	-	-
**l-carnitine**	-		-	-	-	-	1.24	1.24	1.24
**Osmolarity** ***(mOsmol/L)***	284		365	351.9	404.5	486.2	351.9	404.5	486.2
**pH**	5–6		6.6	5.5 ± 0.5	5.5 ± 0.5	5.5 ± 0.5	5.5 ± 0.5	5.5 ± 0.5	5.5 ± 0.5

## Abbreviations

PD	Peritoneal Dialysis
ESRD	End-Stage Renal Disease
CAPD	Continuous Ambulatory PD
APD	Automated PD
PM	Peritoneal Membrane
UF	Ultrafiltration
EMT	Epithelial to Mesenchymal Transition
AA	Amino Acids
HMC	Human Mesothelial Cells
TEER	TransEpithelial Electrical Resistance
GDP	Glucose Degradation Products
Ach	Acetylcholine
OCTN1	Organic Cation Transporter
